# Enlarging blood cyst with atrial septal defect causing tricuspid obstruction

**DOI:** 10.1007/s11748-019-01281-6

**Published:** 2019-12-26

**Authors:** Yu-Lien Chang, Kuo-Sheng Liao, Hsiu-Hsueh Tseng, Yin-Tso Liu

**Affiliations:** 1 Division of Cardiovascular Surgery, Department of Surgery, Taoyuan General Hospital, Ministry of Health and Welfare, No. 1492, Zhongshan Rd., Taoyuan District, Taoyuan, 33004 Taiwan, ROC; 2 Department of Pathology, Taoyuan General Hospital, Ministry of Health and Welfare, Taoyuan, Taiwan, ROC; 3 Department of Education and Research, Taoyuan General Hospital, Ministry of Health and Welfare, Taoyuan, Taiwan, ROC; 4 Division of Cardiovascular Surgery, Department of Surgery, Asia University Hospital, Taichung, Taiwan, ROC

**Keywords:** Intracardiac blood cyst, Atrial septal defect, Tricuspid obstruction

## Abstract

Blood cysts are benign tumors in nature and seldom need surgical removal. Herein, we report an 86-year-old patient with a blood cyst originating from the fossa ovalis accompanied by an atrial septal defect. The patient used the bizarre sleeping postures, including prostrating and worshiping, to relieve chest tightness. The septal base defect kept the tumor progressively enlarged with time, which contributed to a “ball-valve “effect and caused tricuspid flow obstruction. It was resolved after excision. This case report emphasizes that long-term follow-up and increased awareness are required for unpredictable intracardiac blood cyst development.

**Electronic supplementary material:**

The online version of this article (10.1007/s11748-019-01281-6) contains supplementary material, which is available to authorized users.

## Introduction

Since the first report by Elsasser in 1844, intracardiac blood cysts have been believed to be rare primary tumors in adults [1]. Common locations have been reported all over the endocardium of the heart, including the mitral, tricuspid, and pulmonary valves as well as papillary muscles [2, 3]. Blood cysts from both ventricles and the atrial septum in the right atrium (RA) are seldom observed. Furthermore, blood cyst originating from the fossa ovalis accompanied by atrial septal defect (ASD) is extremely rare. ASD nearby the fossa ovalis is a true septal defect, formed by incomplete atrial septation and incomplete covering of the secondary septum. We report an old patient who had a blood cyst popped out from the fossa ovalis in RA. The ASD kept the cyst enlarging with time, subsequently causing obstruction of tricuspid flow.

## Case

**Fig. 1:**
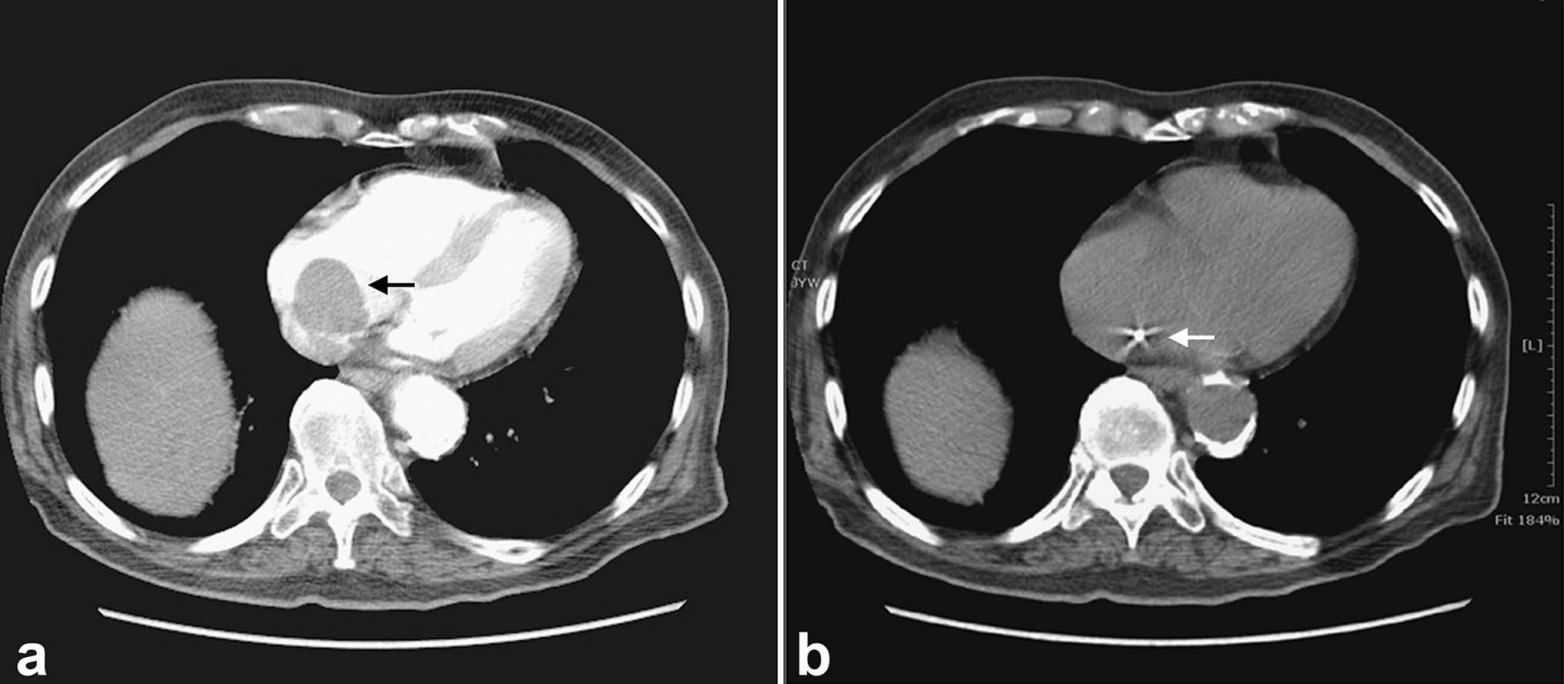
Computed tomographic views showing a space-occupying lesion popping up from the septum (black arrow) in the phase with contrast (**a**) and a pearl-like calcification (white arrow) in the phase without contrast (**b**)

**Fig. 2:**
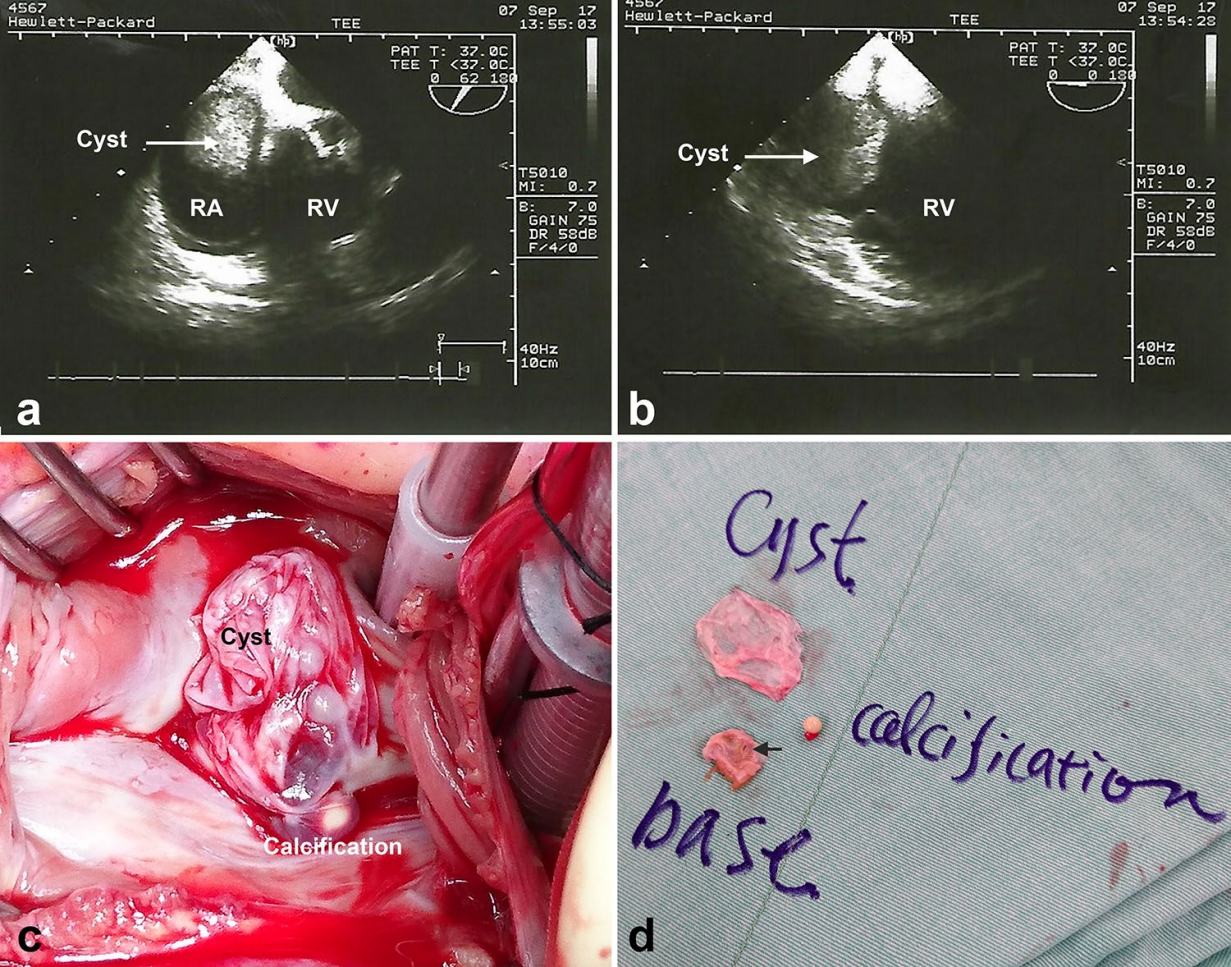
Transthoracic echocardiographic and surgical findings. An oscillating cyst from the septal wall, prolapsing away from the atrioventricular valve in systole (**a**) and blocking it at the end of diastole (**b**) (RV: right ventricle; RA: right atrium). The blood cyst originating from the fossa ovalis with a pinhole-sized defect (black arrow) (**c**, **d**)

An 86-year-old male presented with progressive weakness, chest tightness, exertional dyspnea, and poor appetite in the past 2–3 months. According to his son, the patient could hardly lie down on the bed for the past 2–3 years. Instead, he always kneeled on the bed with his head leaning against his hands to relieve chest tightness to gain a few hours of sleep. The patient was a mason before his retirement in his 70s. There was no systemic disease nor other cardiac history prior to this admission. Nothing remarkable could be mentioned in the medical history and routine examinations. Echocardiography revealed a fibrotic lesion, about 3–4 cm in diameter, occupying the RA, suspected as thrombus. There was no interatrial shunt mentioned. Contrast-enhanced computed tomography (CT) showed a round filling defect about 3.7 cm in diameter adhering to the RA septum (Fig. 1a). In the phase without contrast, a radiopaque spot was found on the septal wall (Fig. 1b). Transesophageal echocardiography (TEE) revealed an oscillating tumor popping out from the atrial septum, which prolapsed away from the atrioventricular (AV) valve in systole and blocked it at the end of diastole, creating a “ball-valve effect” on the tricuspid flow (Fig. 2a, b).

Due to valvular obstruction, tumor excision was performed under the support of cardiopulmonary bypass. Cannulation was built up via aortic arterial line and bicaval venous cannulation. The myocardium was protected by intermittent cold cardioplegia and mild hypothermia to 30 °C. After the aorta was cross-clamped and the heart was vented, the atrium was incised. Out of our expectation, there was no solid tumor in the atrium but a collapsed cyst located on the edge of the fossa ovalis (Fig. 2c). A pearl-like calcification was found within the cyst. When the cyst was removed, the cobblestone-like atrial septal base was exposed. There were several dimples over the base (Fig. 2d). When we pressed the left ventricle, blood could be squeezed out from the pinhole-sized defect of the base, suggesting a small ASD (Video online). To prevent development of other small dimples into the left to right shunts in the future, we gave up direct closure of the hole. Instead, we excised the whole base of the cyst and repaired with a 2.0 × 2.0-cm polytetrafluroethelene patch. The histopathologic report revealed an empty cyst lined by endothelium, which was indicated by a CD34-positive staining. It was 3.0 × 2.0 × 0.2 cm in size and contained non-organized blood. On the other side, a grayish white elastic soft nodule composed of degeneration and calcification was found (Fig. S1). Blood cyst was diagnosed.

The postoperative recovery was uneventful. The patient was transferred to ward from intensive care unit 3 days after the operation and was discharged 2 weeks later. The patient could lie down to sleep ever since the operation and regained his viability 3 months postoperatively. For 2 years of follow-up, there have been no other events.

## Discussion

Intracardiac blood cysts are rare in adults. They are congenital in pathology, common in infancy, and often gradually regress with age [1]. This case describes an uncommon enlarging blood cyst accompanied by ASD in an 86-year-old male. The bizarre sleeping postures, including prostrating and worshiping, explain the possibility of hemodynamic dysfunction of the heart. The clinical course indicated that the cyst kept enlarging with time and finally caused obstruction of the tricuspid flow. We believe that the shunt from the pinhole-sized defect on the atrial septum let blood from the left heart being pressed continuously into the cyst and kept it enlarging. This case reminds us that clinicians should be highly alert to look out for intracardiac obstruction if a patient presents with prostrating and worshiping postures.

The origin of intracardiac blood cysts has no consensus till now. Blood cysts from the atrial septum have been reported [4]; however, blood cyst coexistence with ASD is extremely rare [5]. To our knowledge, this is the second case that reports blood cyst with a septal defect on the base in adulthoods. One common theory of blood cyst formation suggests that blood is trapped in cervices and is sealed off during the development of valves. Moreover, Seebacher et al*.* suggested that the ASD creates a valve-like mechanism, making part of the septum secundum bulge into a cyst [5]. In our observation, only one of the dimples was patent. The cribriform appearance may imply a processing of primary septal absorption. With incomplete atrial folding to form the secondary septum, the pinhole-sized shunt keeps the cyst enlarging. Furthermore, blood cysts could be found over the endocardium of the heart. ASD combined with a valve-like mechanism does not seem to be untenable for cysts that originated somewhere else. This case report provides an interesting hypothesis that the “bubbling of the endothelium” may progress through the development of atrial septation, AV valve formation and ventricular septation. Subsequently, it may regress gradually after birth. Nevertheless, the role of “bubbling” in embryology and the exact physiology of formation remain uncertain.

The definitive diagnosis of intracardiac blood cysts depends on pathology. Although echocardiography is the most convenient tool for preoperative evaluation and long-term follow-up, it is not the ideal tool for differential diagnosis. In our case, the echolucent character was not well captured due to poor cardiac window. CT with contrast only disclosed a space-occupying lesion with well-defined margin in RA. CT without contrast revealed a radiopaque spot analog to calcification found on the septal wall, implying the possibility of blood cysts even with low incidence. Nevertheless, it remains difficult to distinguish blood cysts from other solid intracardiac tumors, including thrombus, myxoma, hydatid cysts, and infective vegetation. Many diagnostic tools like magnetic resonance imaging can only indirectly speculate the occurrence of intracardiac blood cysts. Only a histopathologic report can directly confirm the diagnosis.

## Conclusion

Conservative treatment is suitable for minor intracardiac blood cysts since they are benign in nature. But surgical excision is needed when they cause hemodynamic dysfunction. Importantly, long-term follow-up is necessary when an intracardiac blood cyst is diagnosed, due to unpredictable development.

## Electronic supplementary material

Below is the link to the electronic supplementary material.

Supplementary file1 (MPG 9760 kb)
